# A Bayesian Network Model for Predicting Post-stroke Outcomes With Available Risk Factors

**DOI:** 10.3389/fneur.2018.00699

**Published:** 2018-09-07

**Authors:** Eunjeong Park, Hyuk-jae Chang, Hyo Suk Nam

**Affiliations:** ^1^Cardiovascular Research Institute, College of Medicine, Yonsei University, Seoul, South Korea; ^2^Department of Cardiology, College of Medicine, Yonsei University, Seoul, South Korea; ^3^Department of Neurology, College of Medicine, Yonsei University, Seoul, South Korea

**Keywords:** stroke, bayesian network, prognostic model, machine learning classification, decision support techniques, imbalanced data

## Abstract

Bayesian network is an increasingly popular method in modeling uncertain and complex problems, because its interpretability is often more useful than plain prediction. To satisfy the core requirement in medical research to obtain interpretable prediction with high accuracy, we constructed an inference engine for post-stroke outcomes based on Bayesian network classifiers. The prediction system that was trained on data of 3,605 patients with acute stroke forecasts the functional independence at 3 months and the mortality 1 year after stroke. Feature selection methods were applied to eliminate less relevant and redundant features from 76 risk variables. The Bayesian network classifiers were trained with a hill-climbing searching for the qualified network structure and parameters measured by maximum description length. We evaluated and optimized the proposed system to increase the area under the receiver operating characteristic curve (AUC) while ensuring acceptable sensitivity for the class-imbalanced data. The performance evaluation demonstrated that the Bayesian network with selected features by wrapper-type feature selection can predict 3-month functional independence with an AUC of 0.889 using only 19 risk variables and 1-year mortality with an AUC of 0.893 using 24 variables. The Bayesian network with 50 features filtered by information gain can predict 3-month functional independence with an AUC of 0.875 and 1-year mortality with an AUC of 0.895. We also built an online prediction service, Yonsei Stroke Outcome Inference System, to substantialize the proposed solution for patients with stroke.

## Introduction

A stroke is the second most common cause of death in the world and a leading cause of long-term disability. Patients with stroke have higher mortality than age- and sex-matched subjects who have not experienced a stroke. It is also reported that strokes recur in 6–20% of patients, and approximately two-thirds of stroke survivors continue to have functional deficits that are associated with diminished quality of life (1). Such disability after stroke can be measured by the modified Rankin scale that categorizes functional ability from 0 to 6 (2–4). To discriminate the effect of clinical treatment for patients with ischemic stroke, a score on the modified Rankin scale 0–2 is widely applied for the indication of functional independence after stroke (2).

There are many prognostic models for the functional outcomes and risk of death after stroke. However, an agreed set of guidelines or reporting for the development of prognostic score models are currently unavailable. In a recent systematic review of clinical prediction models, the discriminative performances of models were still unsatisfactory, with the AUC values ranging from 0.60 to 0.72, which are similar to the predictability of experienced clinicians (5).

The prediction of prognosis needs to employ a variety of statistical, probabilistic, and optimization techniques to learn patterns from large, complex, and unbalanced medical data. This complexity challenges researchers to apply machine learning techniques to diagnose and predict the progress of the disease (6, 7). Machine learning has been expected to dramatically improve prognosis, and certain applications have achieved remarkable results (7). These applications have employed various machine learning techniques including a deep neural network (8), support vector machine (8, 9), decision trees (10), and ensemble methods (11, 12) to classify diseases, level of deficits, and morality. Selecting the optimal solution for a decision problem should consider the unique pattern of a data set and the specific characteristics of the problem (13).

The Bayesian network, a machine learning method, predicts and describes classification based on the Bayes theorem (14). Bayesian networks are widely used in medical decision support for their ability to intuitively encapsulate cause and effect relationships between factors that are stored in medical data (15, 16). With these characteristics of conditional probabilities, the Bayesian network can provide interpretable classifiers by logic inherent in a decision support (17, 18). The parameters and their dependences with conditional probabilities of the Bayesian network can be provided either by experts' knowledge (16, 19) or by automatic learning from data (20, 21). In addition, Bayesian networks can be used to query any given node in the network and are therefore substantially more useful in clinics compared with classifiers built based on specific outcome variables (22).

In this study, our aim was to investigate the usefulness of a machine learning method to forecast functional recovery for independent activities and 1-year mortality in patients with acute ischemic stroke. We also introduced an online inference system for predicting functional independence at 3 months and mortality in 1 year of patients with stroke based on the proposed Bayesian network.

## Materials and methods

### Data set

Subjects for this study were selected from consecutive patients with acute ischemic stroke who had been registered in the Yonsei Stroke Registry over a 6.5-year period (January 2007 to June 2013). The Yonsei Stroke Registry is a prospective hospital-based registry for patients with acute ischemic stroke or transient ischemic attack within 7 days after symptom onset (23).

During admission, all patients were thoroughly investigated for medical history, clinical manifestations, and the presence of vascular risk factors. Every patient was evaluated with 12-lead electrocardiography, chest x-ray, lipid profiles, and standard blood tests. All registered patients underwent brain imaging studies including brain computed tomography (CT) and/or MRI. Angiographic studies using CT angiography, magnetic resonance angiography, or digital subtraction angiography were included in the standard evaluation. Additional blood tests for coagulopathy or prothrombotic conditions were performed in patients younger than 45 years. Transesophageal echocardiography was included in the standard evaluation, except in patients with decreased consciousness, impending brain herniation, poor systemic condition, inability to accept an esophageal transducer because of swallowing difficulty or tracheal intubation, or lack of informed consent (24). Transthoracic echocardiography, heart CT, and Holter monitoring were also performed in selected patients (25). When a patient was admitted more than twice because of recurrent strokes, only data for the first admission were used for this study. Initial stroke severity was determined by National Institute of Health Stroke Scale (NIHSS) scores and score tertiles were used for the analysis.

Hypertension was defined as resting systolic blood pressure ≥140 mm Hg or diastolic blood pressure ≥90 mm Hg after repeated measurements during hospitalization or currently taking antihypertensive medication. Diabetes mellitus was defined as fasting plasma glucose values ≥7 mmol/L or taking an oral hypoglycemic agent or insulin. Hyperlipidemia was diagnosed as a fasting serum total cholesterol level ≥6.2 mmol/L, low-density lipoprotein cholesterol ≥4.1 mmol/L, or currently taking a lipid-lowering drug after a hyperlipidemia diagnosis. A current smoker was defined as an individual who smoked at the time of stroke or had quit smoking 1 year before treatment (26). The collection of variables during admission including clinical, imaging, and laboratory data were used in statistical analysis and Bayesian network modeling.

Stroke classification was determined during weekly conferences based on the consensus of stroke neurologists. Data including clinical information, risk factors, imaging study findings, laboratory analyses, and other special evaluations were collected. Along with these data, prognosis during hospitalization and long-term outcomes were also determined. Data were entered into a web-based registry. Stroke subtypes were identified according to the Trial of ORG 10172 in Acute Stroke Treatment (TOAST) classification (27).

For target variables in classification, we collected the outcome variables for patients who were followed in the outpatient clinic or by a structured telephone interview at 3 months and every year after discharge. Short-term functional outcomes at 3 months were determined based on the modified Rankin scale. Major disability was defined as a score on the modified Rankin scale of 3–6, as a poor outcome at 3 months after stroke. Deaths among subjects from January 2001 to December 31, 2013, were confirmed by matching the information in the death records and identification numbers assigned to the subjects at birth (5). We obtained data for the date and causes of death from the Korean National Statistical Office, which were identified based on death certificates (28, 29). The institutional review board of Severance Hospital, Yonsei University Health System, approved this study and waived the patients' informed consent because of a retrospective design and observational nature of this study.

### Bayesian networks

The collected data set was used to construct Bayesian networks for predicting post-stroke outcomes. We extracted a total of 76 random variables of each instance for patient data. A Bayesian network consists of a directed acyclic graph whose nodes represent random variables and links express dependences between nodes. Suppose random variables *V*_*i*_ ∈ *V*(1 ≤ *i* ≤ *n*). A Bayesian network is described as a directed acyclic graph *G* = (*V, A, P*) with links *A* ⊆ *V* × *V* and *P* a joint probability distribution. *P*, a joint probability over *V*, is described as

$$\begin{array}{l} P\left(V\right)=\underset{V_{i}\in V}{\prod }P\left(V_{i}|\pi \left(V_{i}\right)\right), \end{array}$$

where π(*V*_*i*_) is the set of parent nodes of *V*_*i*_.

Training Bayesian network classifiers is the process of parameter learning to find optimal Bayesian structures estimating parameter set of *P* that best represents given data set with labeled instances (13). Given a data set *D* with variable *V*_*i*_, the observed distribution *P*_*D*_ is described as a joint probability distribution over *D*. The learning process now measures and compares the quality of Bayesian networks to evaluate how well the represented distribution explains the given data set. The log-likelihood is the basic common value used for measuring the quality of a Bayesian network as follows:

$$\begin{array}{l} LL\left(B|D\right)=\sum_{V_{i}}log\left(P\left(V_{i}|\pi_{B}\left(V_{i}\right)\right)\right), \end{array}$$

where B is the Bayesian network over *D* and |π_*B*_(*V*_*i*_) is parent nodes of *V*_*i*_ in B(13, 30).

Diverse quality measurement methods have been investigated (31). The algorithm searched the best Bayesian network based on the Bayesian information criterion (32), Bayesian Dirichlet equivalence score (19), Akaike information criterion (AIC) (33), and the maximum description length (MDL) scores (30, 34). In this study, we used the MDL score to evaluate the quality of a Bayesian network. The MDL score is described as

$$\begin{array}{l} MDL=-LL\left(B|D\right)+\frac{logN}{2}\cdot |B|, \end{array}$$

where *N* is the number of instances in *D*, and |B| is the number of parameters in B. The smaller the MDL score, the better the network. The search algorithm, greedy hill-climbing algorithm (35) in our study, selects the best Bayesian network by calculating MDL scores of candidate networks. For the type of Bayesian network structure, we constructed tree-augmented network (TAN) structures that restrict the number of parents to two nodes (36).

### Prediction process

**Figure 1:**
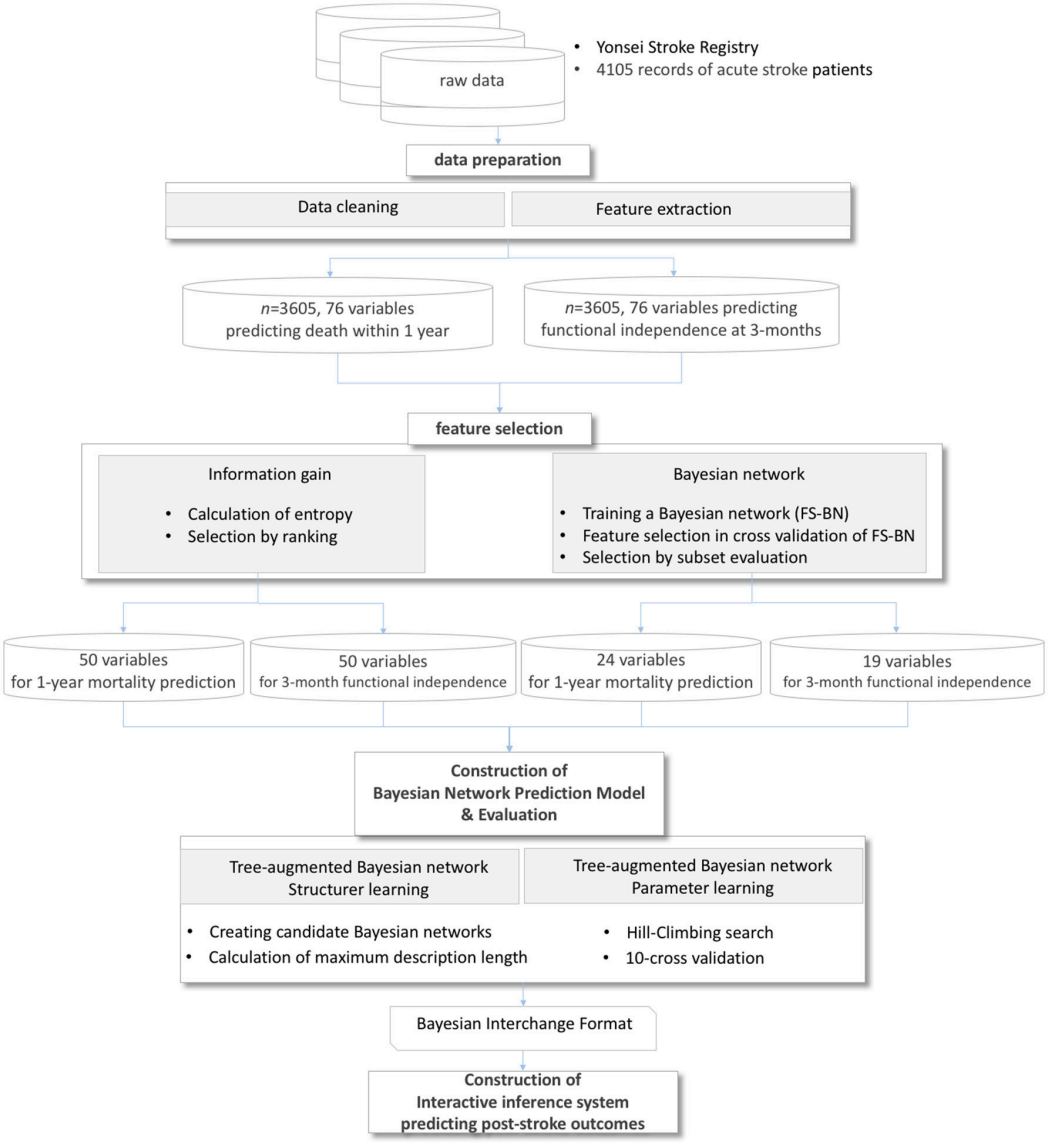
Process of a prediction system for post-stroke outcomes.

The entire process of a Bayesian network-based prediction system is shown in Figure 1. A total of 76 features were extracted from the Yonsei Stroke Registry and data preparation process filtered records with missing outcome variables and exclusion criteria. For feasible prediction service in clinical environment, we performed two different feature selection methods.

Feature selection or dimension reduction is the process of reducing the number of random variables under consideration by obtaining a set of principal variables (37, 38). Feature selection improves the overfitting problem caused by irrelevant or redundant variables that may strongly bias the performance of the classifier. The definition of feature selection in formal expression is described in Drugan and Wiering (30) and Hruschka et al. (39). In many studies, feature selection methods are categorized into filters, wrappers, or embedded methods that are applied to the data set in advance of the training learning algorithm, or to embed feature selection in the learning process (37, 40). Filter methods select features based on a performance measure regardless of the employed data modeling algorithm. The filter approach selects random variables based on information gain score, ReliefF, or correlation-based method by ranking variables or searching subset of variables. Information gain measures the amount of entropy as a measure of uncertainty reduced by knowing a feature (41–43); ReliefF evaluates the worth of an attribute by repeatedly sampling an instance and considering the value of the given attribute for the nearest instance of the same and the different class (44, 45); and correlation evaluates the worth of a subset of attributes by considering the individual predictive ability of each feature along with the degree of redundancy between them (46, 47). Unlike the filter approach, wrapper methods measure the usefulness of a subset of features by actually training a model on it. We evaluated the performance of Bayesian networks with a reduced variable set selected by information gain and Bayesian network algorithms that are popular in filter and wrapper methods (42, 48, 49).

First, we tested the Bayesian network classifier with features chosen by information gain based on entropy of each feature. The other feature selection method, considering the characteristics of Bayesian network classifiers, reduces the variable set by evaluating the performance of the Bayesian network classifier in cross-validation in which a search algorithm extracts a subset of attributes to maximize AUC in prediction (Figure 1). The optimization for AUC is to solve the imbalance between the number of survival and mortal subjects.

Using the reduced variables by feature selection, the system constructed a Bayesian network prediction model to search optimal Bayesian network structures and parameters. We evaluated the performance of prediction algorithms using (1) a basic tree-augmented Bayesian network, (2) a tree-augmented Bayesian network with features filtered by information gain, and (3) a tree-augmented Bayesian network with features filtered by the wrapper of a Bayesian network. The performances of all Bayesian networks and predictive models were evaluated based on the AUC, specificity, and sensitivity of 10-fold cross-validations (50). We also implemented an online prediction system for post-stroke outcomes embedding the trained classifiers. In the validation process, we bound the minimum sensitivity as 0.50 to utilize the trained classifiers in real-world applications with imbalanced data.

## Results

### Statistical characteristics

During the study period, 4,105 consecutive patients with acute ischemic stroke or transient ischemic attack were registered to the Yonsei Stroke Registry. Exclusion criteria of this study were patients with the stroke subtypes other than cryptogenic stroke including transient ischemic attack (*n* = 326), foreigner (*n* = 48), missing data (*n* = 29), follow-up loss (*n* = 97). After exclusion, a total of 3,605 patients were finally enrolled for this study. The mean age was 65.9 ± 12.6 years, and 60.7% were men. A comparison of demographic characteristics between the outcome at 3 months and death within 1 year is shown at Table 1. Patients with poor outcome were older, more likely to be women, not a current smoker, frequently had old stroke, hypertension, atrial fibrillation, congestive heart failure, peripheral artery obstructive disease, or anemia. Thrombolysis or endovascular mechanical thrombectomy, symptomatic intracranial hemorrhage, and herniation are frequent in patients with poor outcome. Laboratory data showed that patients with poor outcome showed lower hemoglobin, hematocrit, albumin, prealbumin, body weight and higher ESR, fibrinogen, hsCRP, and D-dimer level. The differences of demographics of patients between survival and death within 1 year were similar with functional outcome at 3 months. D-dimer levels were significantly higher in patients who died within 1 year compared with survivors (3079.8 ± 9723.3 vs. 464.5 ± 1759.3, *p* < 0.001).

**Table 1:** Demographic characteristics and comparison of outcome at 3 months and death within 1 year.

	**Total**	**Outcome at 3 months**			**Death within 1 year**		
	**(*N* = 3,605)**	**Good outcome** **(*N* = 2,653)**	**Poor outcome** **(*N* = 952)**	***p***	**No** **(*N* = 3,171)**	**Yes** **(*N* = 434)**	***p***
Age	65.9 ± 12.6	64.0 ± 12.3	71.2 ± 11.9	<0.001	64.8 ± 12.4	73.9 ± 11.2	<0.001
Sex				<0.001			0.016
F	1,416 (39.3%)	969 (36.5%)	447 (47.0%)		1,222 (38.5%)	194 (44.7%)	
M	2,189 (60.7%)	1,684 (63.5%)	505 (53.0%)		1,949 (61.5%)	240 (55.3%)	
Hypertension	2,675 (74.2%)	1,940 (73.1%)	735 (77.2%)	0.015	2,675 (74.2%)	1,940 (73.1%)	0.023
Diabetes	1,144 (31.7%)	827 (31.2%)	317 (33.3%)	0.243	1,144 (31.7%)	827 (31.2%)	0.282
Hypercholesterolemia	747 (20.7%)	554 (20.9%)	193 (20.3%)	0.726	685 (21.6%)	62 (14.3%)	0.001
Current smoking	856 (23.7%)	704 (26.5%)	152 (16.0%)	<0.001	856 (23.7%)	704 (26.5%)	<0.001
Old stroke	472 (13.1%)	301 (11.3%)	171 (18.0%)	<0.001	401 (12.6%)	71 (16.4%)	0.038
Atrial fibrillation	813 (22.6%)	482 (18.2%)	331 (34.8%)	<0.001	623 (19.6%)	190 (43.8%)	<0.001
Coronary artery disease	811 (22.5%)	603 (22.7%)	208 (21.8%)	0.608	717 (22.6%)	94 (21.7%)	0.701
Congestive heart failure	184 (5.1%)	110 (4.1%)	74 (7.8%)	<0.001	134 (4.2%)	50 (11.5%)	<0.001
Peripheral artery obstructive disease	110 (3.1%)	60 (2.3%)	50 (5.3%)	<0.001	85 (2.7%)	25 (5.8%)	0.001
Initial NIHSS score	5.6 ± 6.3	3.4 ± 4.0	11.5 ± 7.5	<0.001	4.8 ± 5.4	11.5 ± 8.4	<0.001
TOAST				<0.001			<0.001
LAC	321 (8.9%)	285 (10.7%)	36 (3.8%)		312 (9.8%)	9 (2.1%)	
LAA	741 (20.6%)	504 (19.0%)	237 (24.9%)		661 (20.8%)	80 (18.4%)	
CE	991 (27.5%)	688 (25.9%)	303 (31.8%)		823 (26.0%)	168 (38.7%)	
SOD	89 (2.5%)	68 (2.6%)	21 (2.2%)		80 (2.5%)	9 (2.1%)	
UT	668 (18.5%)	498 (18.8%)	170 (17.9%)		587 (18.5%)	81 (18.7%)	
UN	785 (21.8%)	607 (22.9%)	178 (18.7%)		703 (22.2%)	82 (18.9%)	
UI	10 (0.3%)	3 (0.1%)	7 (0.7%)		5 (0.2%)	5 (1.2%)	
Anemia	617 (17.1%)	361 (13.6%)	256 (26.9%)	<0.001	450 (14.2%)	167 (38.5%)	<0.001
Thrombolysis	485 (13.5%)	272 (10.3%)	213 (22.4%)	<0.001	377 (11.9%)	108 (24.9%)	<0.001
Symtomatic ICH	92 (2.6%)	10 (0.4%)	82 (8.6%)	<0.001	43 (1.4%)	49 (11.3%)	<0.001
Herniation	105 (2.9%)	3 (0.1%)	102 (10.7%)	<0.001	38 (1.2%)	67 (15.4%)	<0.001
Body weight	62.9 ± 11.1	64.0 ± 10.9	60.0 ± 11.2	<0.001	63.6 ± 11.0	57.8 ± 10.8	<0.001
hgb	13.8 ± 2.0	14.0 ± 1.9	13.3 ± 2.2	<0.001	14.0 ± 1.9	12.7 ± 2.3	<0.001
hct	40.6 ± 5.6	41.1 ± 5.3	39.3 ± 6.1	<0.001	41.0 ± 5.3	37.9 ± 6.5	<0.001
esr	23.9 ± 22.2	21.2 ± 20.1	31.3 ± 25.8	<0.001	22.1 ± 20.6	36.5 ± 28.8	<0.001
pt	1.0 ± 0.5	1.0 ± 0.3	1.0 ± 0.7	0.123	1.0 ± 0.5	1.0 ± 0.2	0.002
Albumin	4.2 ± 0.5	4.3 ± 0.4	4.0 ± 0.5	<0.001	4.3 ± 0.4	3.9 ± 0.6	<0.001
Prealbumin	223.7 ± 72.6	239.0 ± 69.9	205.6 ± 71.6	<0.001	233.3 ± 69.8	186.8 ± 71.4	<0.001
Fibrinogen	322.8 ± 94.3	316.1 ± 83.9	341.5 ± 116.8	<0.001	320.1 ± 88.5	342.5 ± 128.5	0.001
hsCRP	11.3 ± 48.4	7.5 ± 49.7	22.2 ± 42.7	<0.001	9.2 ± 48.4	27.3 ± 45.5	<0.001
D-dimer	779.0 ± 3846.1	418.4 ± 1704.4	1788.2 ± 6834.6	<0.001	464.5 ± 1759.3	3079.8 ± 9723.3	<0.001

### Structure and parameters of bayesian networks

**Figure 2:**
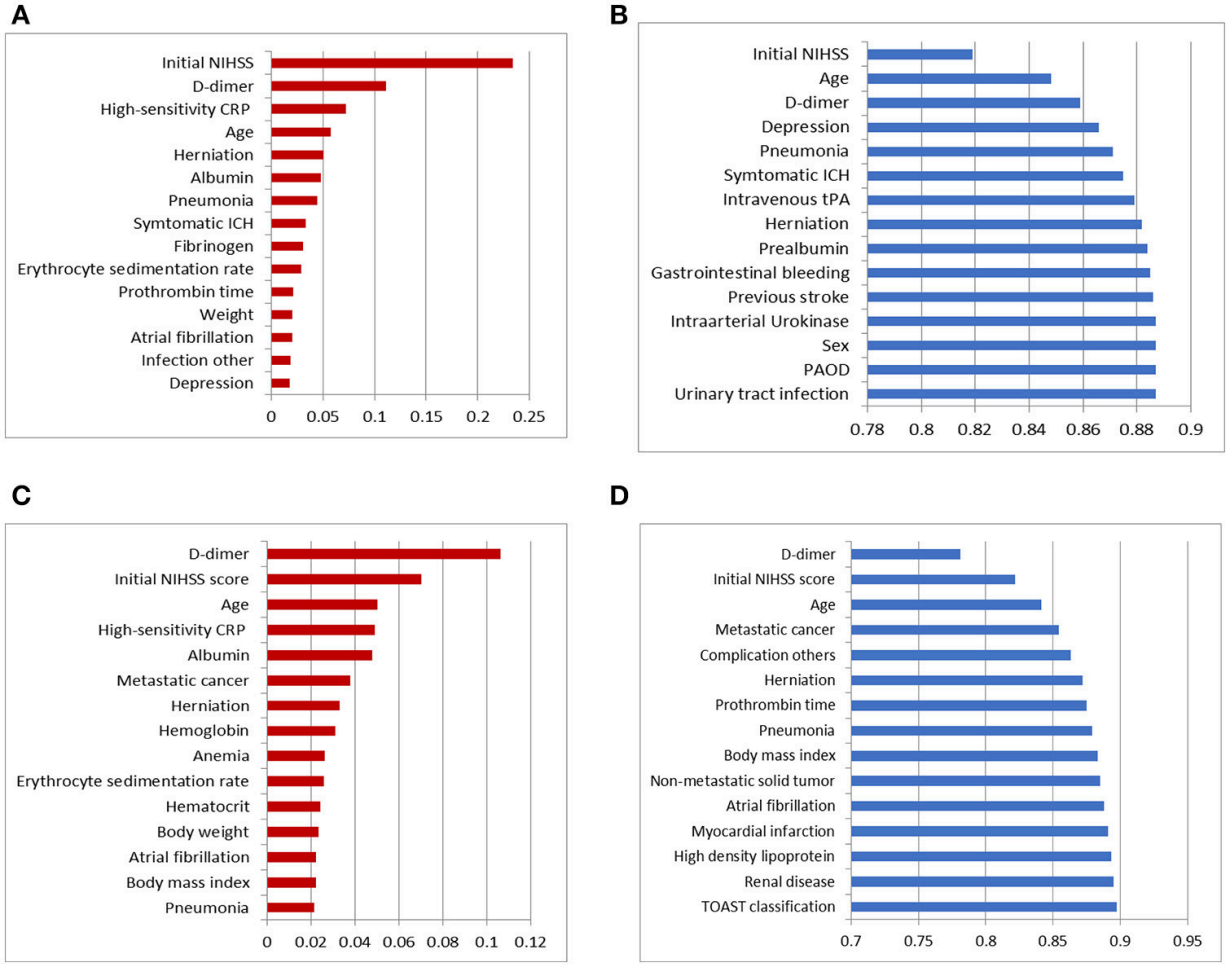
Top 15 variables in dimension reduction for post-stroke outcome prediction: **(A)** variables filtered by ranks of information gain for predicting functional independence at 3 months, **(B)** variables selected by the wrapper of the Bayesian network classifier with greedy subset selection for predicting functional independence at 3 months, **(C)** variables filtered by ranks of information gain for predicting 1-year mortality, and **(D)** variables selected by the wrapper of the Bayesian network classifier with greedy subset selection for predicting 1-year mortality.

As we described in Figure 1, two different feature selection techniques were performed in our experiment: variables selected by information gain with ranking or variables selected by a wrapper embedding Bayesian network with greedy stepwise subset selection in cross-validation. The top-ranked variables in the filter by information gain and the wrapper of the Bayesian network in forecasting functional independence at 3 months are shown in Figures 2A,B, and variables for predicting 1-year mortality are shown in Figures 2C,D. The most affective factor for functional recovery prediction was Initial NIHSS, while D-dimer ranked top in 1-year mortality prediction. The common variables for predicting post-stroke outcomes were Initial NIHSS, D-dimer, hsCPR, and Age. However, the subset-searching algorithm selects a method differently from the ranking method that evaluates the individual variables separately; thus, certain variables were excluded from the selected subset even though their ranks are high in individual evaluation.

**Figure 3:**
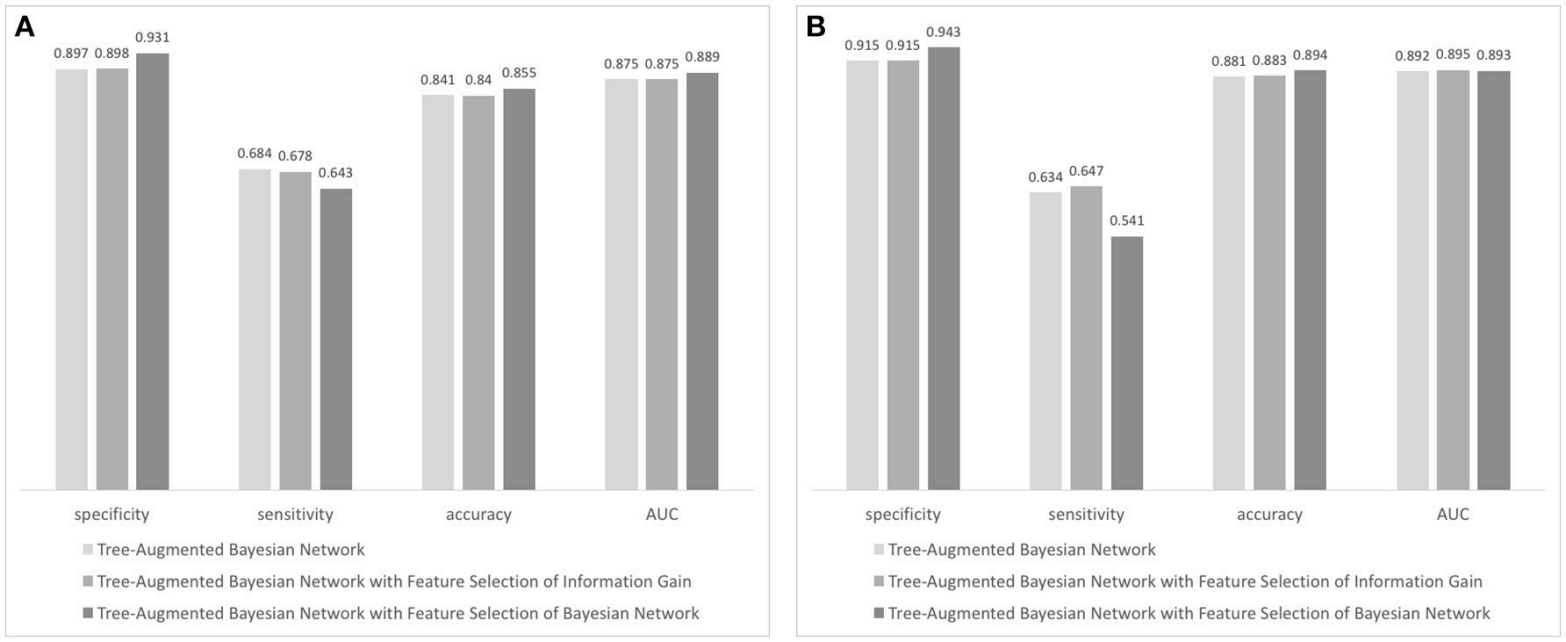
Performance evaluation of Bayesian network-based classifiers: **(A)** performance of classifiers forecasting 90-day functional independence and **(B)** performance of classifiers for 1-year mortality prediction.

Using the result of feature selection, we trained three tree-augmented Bayesian network classifiers; (1) Tree-augmented Bayesian network with the entire dataset, (2) tree-augmented Bayesian network with features filtered by ranking of information gain, and (3) tree-augmented Bayesian network with features filtered by the wrapper of the Bayesian network classifier (see Figure 3). The predictive performance for 3-month outcomes is shown in Figure 3A. The classifier trained with features chosen by the Bayesian network's subset evaluation performs in prediction of 3-month functional recovery with the specificity of 0.931, accuracy of 0.643, and AUC of 0.889 (95% CI, 0.879–0.899) although the sensitivity (0.643) is slightly lower than other algorithms. The tree-augmented Bayesian network without feature selection achieved the AUC of 0.875 (95% CI, 0.864–0.886), but the highest sensitivity of 0.684; and the Bayesian network with features by ranking of information gain obtained the AUC of 0.875 (95% CI, 0.864–0.886) and mid-level performance between two other algorithms. The Bayesian network classifier with feature selection achieved best performance in most metrics except sensitivity, although it reduced the variable set from 76 variables to 19 variables, resulting in a great reduction in model construction time.

**Figure 4:**
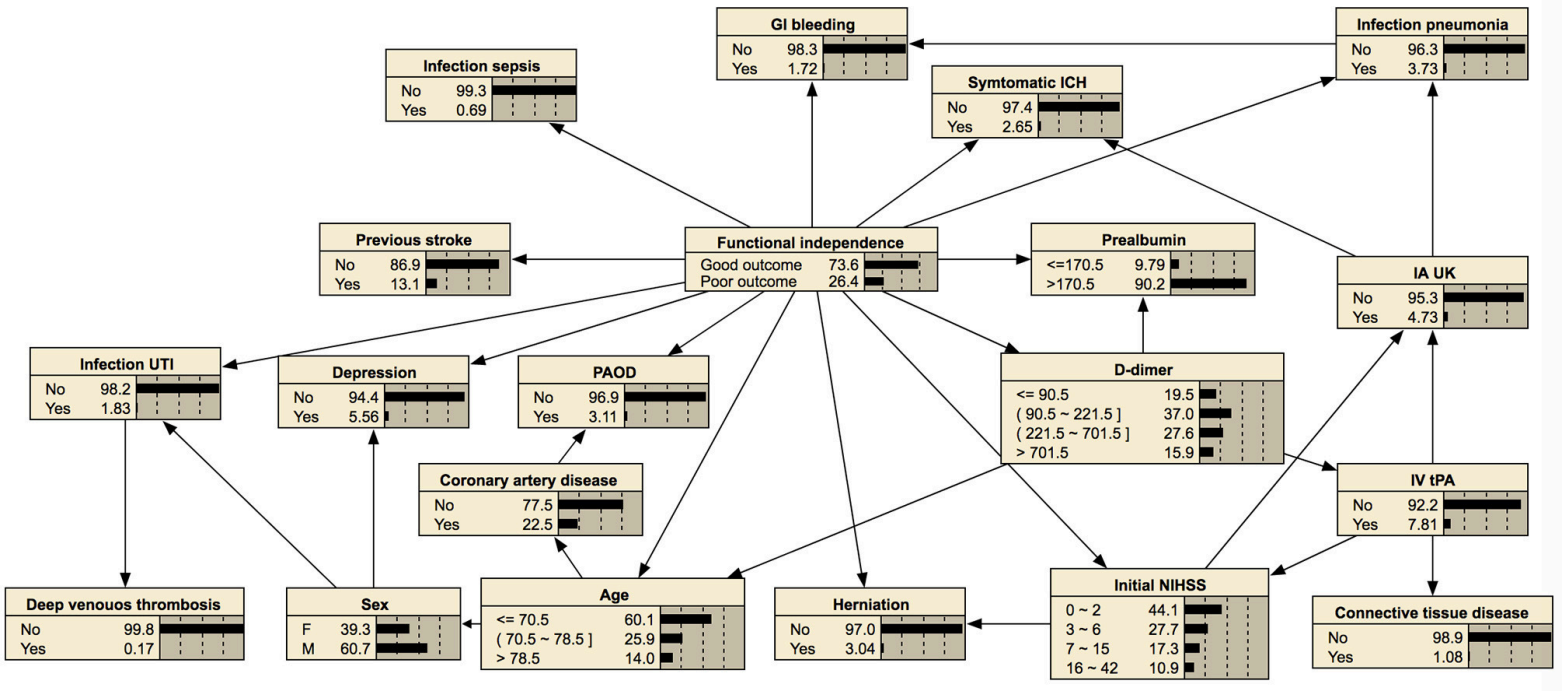
Bayesian network for predicting functional independence at 3 months. The tree-augmented Bayesian network used 19 variables selected by the wrapper of the Bayesian network for prediction.

**Figure 5:**
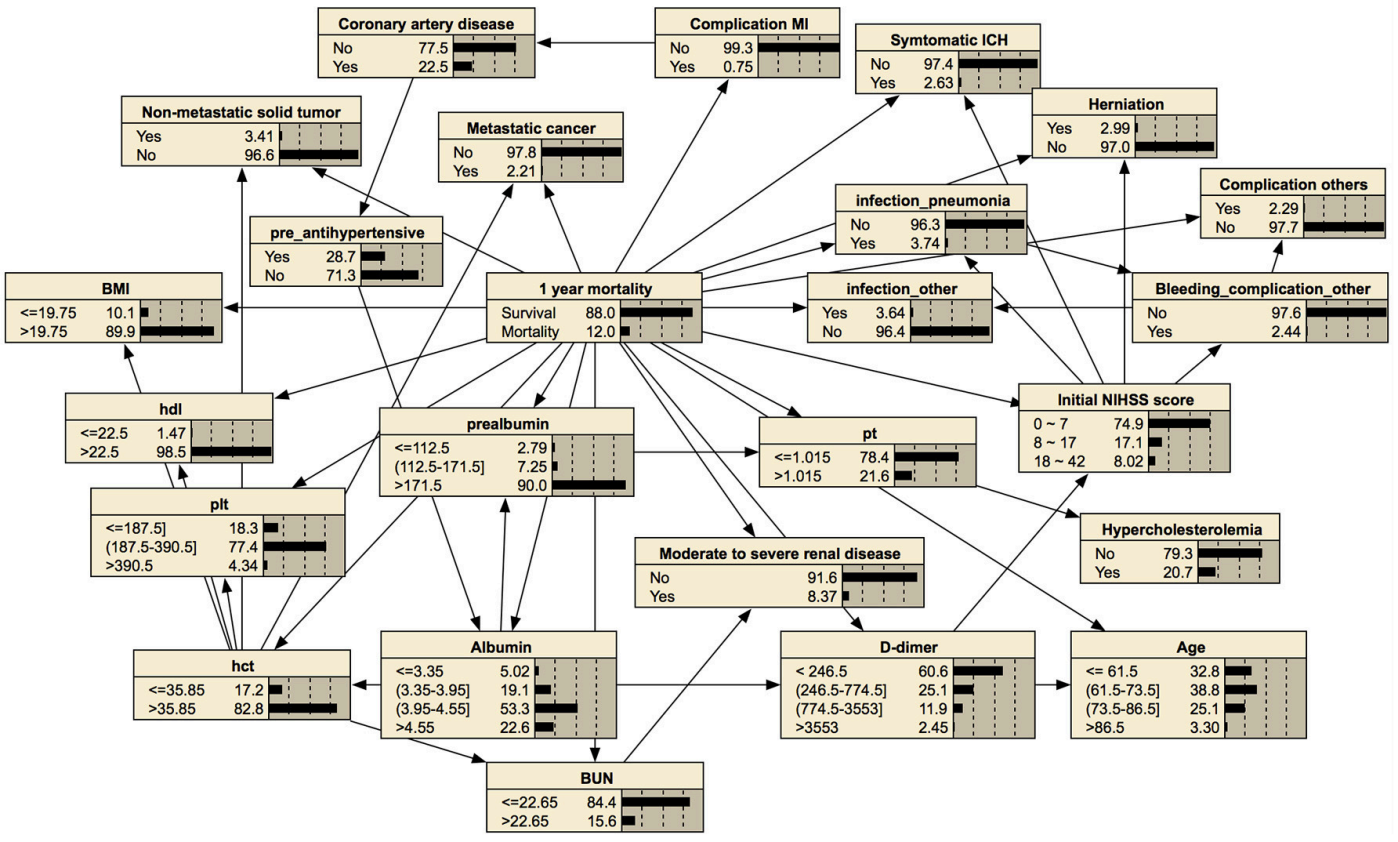
Bayesian network for predicting 1-year mortality. The tree-augmented Bayesian network used 24 variables selected by the wrapper of the Bayesian network for prediction.

In prediction of 1-year mortality, AUCs of three algorithms were not significantly different (0.892 with 95% CI, 0.872–0.912; 0.895 with CI, 0.875–0.915; and 0.893 with CI, 0.873–0.913). All algorithms achieved higher specificities in predicting 1-year mortality than those for the prediction of functional independence (0.915 vs. 0.897 with a basic Bayesian network, 0.915 vs. 0.898 with a Bayesian network with features filtered by information gain, and 0.943 vs. 0.931 with a Bayesian network with features chosen by the wrapper of the Bayesian network classifier). The Bayesian network algorithm with feature selection for 1-year mortality cuts out the entire variable set to 24 variables that curtail network construction time. The final Bayesian networks predicting functional recovery and 1-year mortality are shown in Figures 4, 5, respectively.

### Online interactive system for predicting post-stroke outcomes

**Figure 6:**
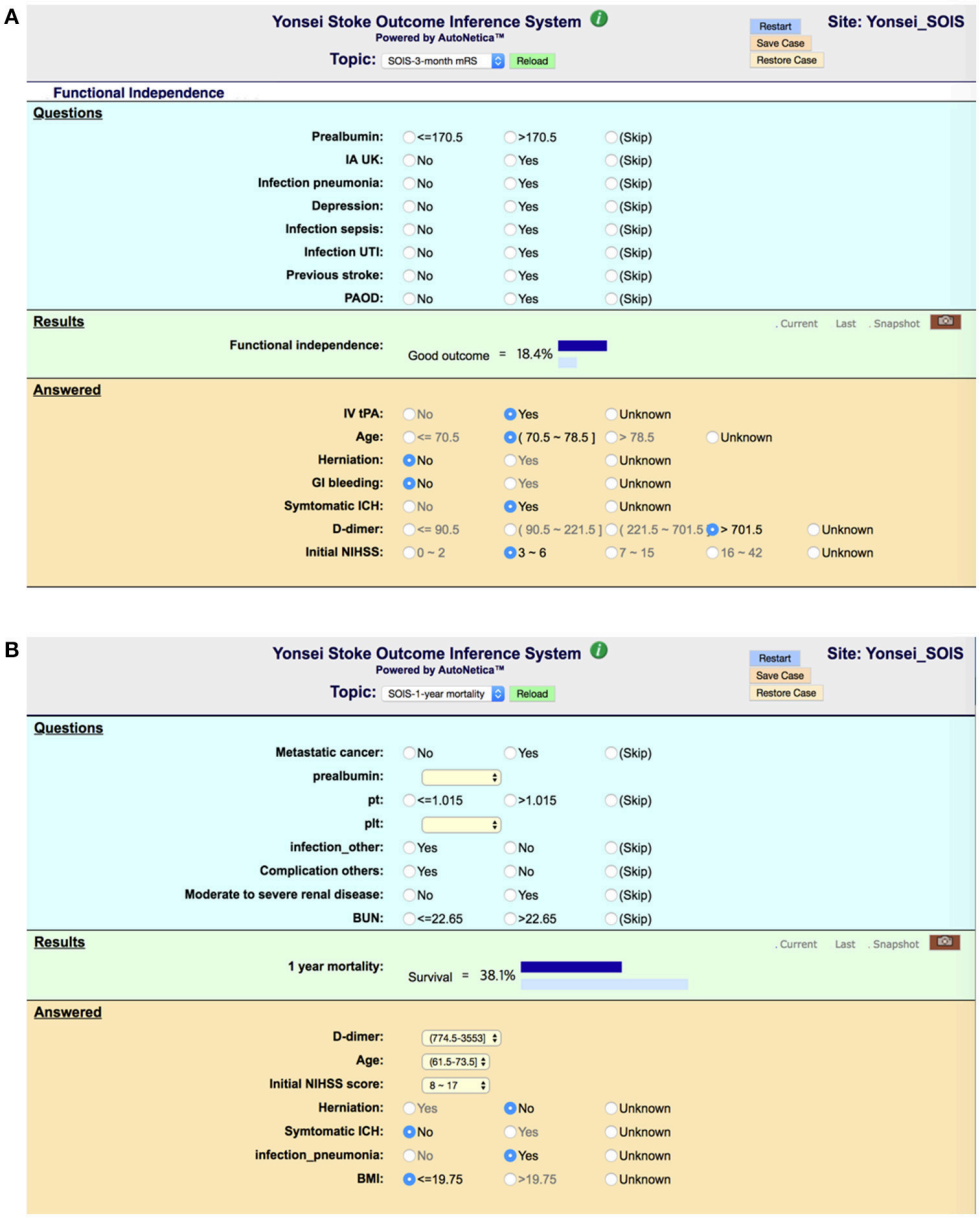
Screenshots of an online prediction system, Y-SOIS (Yonsei Stroke Outcome Inference System). **(A)** Y-SOIS forecasts the functional independence at 3 months and **(B)** Y-SOIS forecasting the 1-year mortality.

To realize decision support using Bayesian network classifiers, we embedded our final Bayesian networks into an online inference system, Y-SOIS (Yonsei-Stroke Outcome Inference System, https://www.hed.cc/?a=Yonsei_SOIS), that enables answering post-stroke outcomes when users provide available risk variables. Figure 6 shows the screenshots of Y-SOIS.

## Discussion

Interpretability is a core requirement for machine learning models in medicine, because both patients and physicians need to understand the reason behind a prediction (51). This study presents an evaluation of Bayesian networks in providing post-stroke outcomes estimates based on the collected demographic data, lab result, and initial neurological assessment. The stroke-specific variables were selected from a large stroke registry, and our experiment filtered those variables into the Bayesian network-suitable reduced set. The trained Bayesian networks were embedded in our online prediction system.

### Strength of a bayesian network on stroke outcome measurements

Research on stroke outcomes is essential for both clinical care and policy development, because approximately two-thirds of stroke survivors continue to experience functional deficits and approximately 1 of 10 patients died within 1 year (5). The prediction of post-stroke outcomes thus requires high accuracy in classification along with the understandable result that can be explained to patients. A Bayesian network can intuitively make connections between variables in medical data and provide interpretable determination in medical decision (17, 18). Therefore, Bayesian networks are well suited for representing uncertainty and causality in prediction for patients with stroke. In recent machine learning studies, a Bayesian neural network is focused on a state of the art method which estimates predictive uncertainty (52). In Kendall and Gal (53), a Bayesian deep learning framework combines input-dependent aleatoric uncertainty together with epistemic uncertainty, to solve the black-box problem in deep learning. Constructing Bayesian networks enables medical diagnosis or prediction with incomplete and partially correct statistics, because it determines causes and effects based on the conditional probability between variables (54).

### Prediction with imbalanced data

Often real-world data sets are predominately composed of normal instances with only a small percentage of interesting instances; therefore, class imbalance is one of the most important challenges (55). Our study also has heavily unbalanced classes in mortality prediction (3,171:434). Suppose entire positive instances were classified into negative class; then the accuracy is 0.880 in 1-year mortality prediction, although mortality is not predicted at all. Most machine learning algorithms train classifiers mainly searching for higher accuracy; therefore, the minority class is less considered in the training process. To challenge this imbalanced classification, a number of techniques have been proposed (56): oversampling approaches create minority instances by simple duplication or synthetic-minority oversampling technique (SMOTE) (57–59); certain classifiers with undersampling beat oversampling (60); cost-sensitive methods weigh higher penalty on misclassification of the minority class (61); and bagging, boosting, and hybrid approaches utilize feedback from misclassification in previous stages of learning (62).

In addition to the capability of interpretable prediction and reduced uncertainty, a Bayesian network is strong machine learning in classifying an imbalanced data set as investigated in Drummond and Holte (60) and Monsalve-Torra et al. (63). In Monsalve-Torra et al. (63), the Bayesian network outperformed radial basis function and multilayer perceptron in sensitivity. In our experiment, the learning process searched the best Bayesian network structure and parameters for the highest AUC while it guarantees at least 0.5 in sensitivity. A more computation-expensive searching algorithm such as repeated hill climbing might be helpful to increase sensitivity in classification.

### Visualized probability of outcomes after stroke

Bayesian networks can also provide a visual graph structure. We constructed a tree-augmented Bayesian network structure that shows an association between nodes. This visualization of conditional probability might be helpful for clinical reasoning. For example, a Bayesian network can provide the association among symptomatic intracranial hemorrhage, higher initial NIHSS score, or higher 1-year mortality with conditional probability, as shown in Figure 5. Therefore, our prediction model of post-stroke outcomes differs from the black-box concept of other machine learning methods (54).

The reduction of dimension is also helpful to visualize inference of prediction. The results demonstrated that the Bayesian network classifier with a reduced variable set can adapt the size of a network for better interpretability with a minimal or better impact on other performance.

### Predictors of post-stroke outcomes

In this study, the information gain analysis showed that “D-dimer” was the highest feature in predicting 1-year mortality. We previously reported that a high D-dimer level by itself appeared to be associated with an increased risk of mortality (64). D-dimer can be found to be elevated in various thrombotic and inflammatory conditions, including ischemic heart disease, infection, or malignancy. These conditions are frequently found in patients with stroke and can increase the risk of mortality (65). However, patients with comorbid diseases were frequently excluded from the clinical trials, so there are no guidelines and evidence whether to treat or not patients with serious comorbid diseases in real clinical practice. In this respect, providing information of the impact of the comorbid condition with a Bayesian network might be helpful to predict the outcomes.

## Limitations and future direction

This study was conducted in a single university hospital and focused on those of East Asian descent. To provide generalizability on our prediction system, we will include various cohorts including different ethnics or patients who received thrombolysis or endovascular thrombectomy. We have plan to apply the interpretable prediction for the SECRET (SElection CRiteria in Endovascular thrombectomy and Thrombolytic therapy) study, which is a nationwide registry for hyperacute stroke. Consecutive patients who received intravenous thrombolysis and/or endovascular thrombectomy were registered (Clinical Trial Registration: NCT02964052). Bayesian network analysis of this specific condition can be used to predict outcome in patients with hyperacute stroke. We will also enlarge our training data including data of various populations by applying the proposed solution to global data archives. Additive risk predictors might be selected as determinant features in a Bayesian network, and it makes the prediction system more applicable in a global clinical environment.

## Author contributions

HN designed the study; EP analyzed the data and wrote the manuscript; and H-jC and HN contributed to data interpretation and revising the manuscript.

### Conflict of interest statement

The authors declare that the research was conducted in the absence of any commercial or financial relationships that could be construed as a potential conflict of interest.
